# Controlling Human Rabies: The Development of an Effective, Inexpensive and Locally Made Passive Cooling Device for Storing Thermotolerant Animal Rabies Vaccines

**DOI:** 10.3390/tropicalmed5030130

**Published:** 2020-08-11

**Authors:** Ahmed Lugelo, Katie Hampson, Machunde Bigambo, Rudovick Kazwala, Felix Lankester

**Affiliations:** 1 Environmental Health and Ecological Sciences Department, Ifakara Health Institute, 78373 Dar es Salaam, Tanzania; 2 Boyd Orr Centre for Population and Ecosystem Health, Institute of Biodiversity, Animal Health and Comparative Medicine, University of Glasgow, Glasgow G12 8QQ, UK; Katie.Hampson@glasgow.ac.uk; 3 Department of Veterinary Medicine and Public Health, Sokoine University of Agriculture, 3105 Morogoro, Tanzania; kazwala@suanet.ac.tz; 4 Global Animal Health Tanzania, 1642 Arusha, Tanzania; bigambochunde@gmail.com (M.B.); felix.lankester@wsu.edu (F.L.); 5 Paul G. Allen School for Global Animal Health, Washington State University, Pullman, WA 99164, USA

**Keywords:** dog-mediated rabies, mass vaccination, thermotolerance, rabies control, co-design

## Abstract

Thermotolerant vaccines greatly improved the reach and impact of large-scale vaccination programs to eliminate diseases such as smallpox, polio and rinderpest. A recent study demonstrated that the potency of the Nobivac^®^ Canine Rabies vaccine was not impacted following experimental storage at 30 °C for three months. We conducted a study to develop a passive cooling device (PCD) that could store thermotolerant vaccines under fluctuating subambient temperatures. Through a participatory process with local communities in Northern Tanzania, we developed innovative PCD designs for local manufacture. A series of field experiments were then carried out to evaluate the effectiveness of five PCDs for vaccine storage under varying climatic conditions. Following iterative improvement, a final prototype “Zeepot Clay” was developed at the cost of US$11 per unit. During a further field-testing phase over a 12-month period, the internal temperature of the device remained below 26 °C, despite ambient temperatures exceeding 42 °C. Our study thus demonstrated that locally designed PCDs have utility for storing thermotolerant rabies vaccines at subambient temperatures. These results have application for the scaling up of mass dog vaccination programs in low-and-middle income countries, particularly for hard-to-reach populations with limited access to power and cold-chain vaccine storage.

## 1. Introduction

Infectious diseases remain a major public health problem, with approximately 43% of global deaths resulting from infectious diseases in low-and-middle income countries (LMICs) [1,2]. In recent years considerable efforts have been invested to tackle these health problems leading to the production of many safe, effective and high-quality vaccines and the implementation of large-scale immunization programs [3,4]. Indeed, immunization has been shown to be a cost-effective way of controlling and even eradicating infectious diseases [5,6]. For example, in 1966 the World Health Organization (WHO) launched the global campaign to eradicate smallpox, which led to the disease being declared eradicated in 1980 [7].

The cold-chain system is a crucial component of successful immunization programs and describes the environment in which vaccines are stored and transported within a recommended temperature range (2–8 °C) from the manufacturer to the point of immunization, with the aim of maintaining the potency of vaccines. The system consists of cold chain equipment, such as cold rooms, refrigerators, freezers and vaccine carriers, as well as temperature monitoring devices. In LMICs, public health and veterinary resources are often limited resulting in difficulties maintaining cold chain conditions and unreliable electric power and poor roads often hinder installation of cold chain equipment [8]. Technological innovations have begun to tackle these challenges. For example, electric-free vaccine storage devices (off-grid refrigerators) have been developed to store vaccines in areas without access to or with unreliable power [9,10]. However, the batteries used in off-grid refrigerators often have limited capacity and are costly to maintain [8,11]. Solar direct-drive refrigerators have been developed to replace battery powered refrigerators [12]. ‘Direct-drive’ technology uses solar energy to freeze cooling media into ice banks, which are then used to keep refrigerators cold. However, these refrigerators are costly to purchase and maintain, limiting their use [13]. Another generation of vaccine storage devices, passive cooling devices (PCDs) [14,15], do not require power (electric, solar or gas) to keep vaccines cool. Rather, PCDs use cooling media such as ice packs or water, and insulation to maintain a cool storage environment [10]. A variety of PCDs are on the market with the ability to keep vaccines cool even in very hot environments, but price remains an obstacle. For example, the ARKTEK™ PCD is able to keep vaccines between 2 and 8 °C for up to 4 weeks, but a single carrier costs US$1200-2400. Less expensive alternatives, affordable in LMICs, are required.

Canine-mediated human rabies is 100% vaccine preventable through both provision of post-exposure prophylaxis for people bitten by rabid dogs and large-scale mass dog vaccination programs to interrupt transmission in domestic dog populations and prevent spill over to humans. Despite this fact, rabies is responsible for over 59,000 deaths every year worldwide, with most occurring in LMICs (Knobel, 2005). Mass dog vaccination (MDV) is the only control measure that offers the prospect of eliminating rabies at the source. However, to implement MDV programs at the scale required to control the disease requires functioning cold chain systems and many rabies endemic countries lack stable power supplies, especially in remote areas. Benefits could therefore be achieved through innovations in the development of thermotolerant vaccines and affordable vaccine storage systems for keeping vaccines below ambient temperatures.

In recent years, efforts have been directed to address the challenges of storing rabies vaccine in remote areas without cold chain infrastructure. A study conducted in 2015 determined that the Nobivac^TM^ Canine Rabies vaccine (MSD Animal Health, Boxmeer, The Netherlands) remained potent following storage at 25 °C for six months and at 30 °C for three months [16]. These findings opened up the possibility of storing these vaccines outside of refrigeration units, an outcome that could benefit rabies control in remote areas where electricity provision is poor. Nonetheless, keeping vaccines in such locations would still require some form of storage and given that temperatures over 30 °C were shown to impact vaccine potency [16], the temperature inside storage units should be as cool as possible. This study describes the development and subsequent testing of a PCD to store rabies vaccines for extended periods in advance of and during MDV campaigns. Key objectives were that the PCD be designed and developed using local knowledge and affordable materials. Given that a commonly used canine rabies vaccine is thermotolerant, the development of an effective PCD, that allows storage of vaccines at subambient temperatures, could support the scaling up of national rabies control and elimination strategies, which are currently being developed across East Africa.

## 2. Materials and Methods

The study, which was conducted in the Mara region in Northern Tanzania, comprised of four phases: PCD design and development, field testing, refinement and final testing.

### 2.1. Phase I: PCD Design and Development

Initially a one-day workshop was held that involved forty-five stakeholders from the Ministry of Livestock and Fisheries Development, the Mara region community, as well as local public health professionals and researchers. The objectives of the workshop were to communicate research findings regarding the determination of rabies vaccine thermotolerance and to allow workshop delegates to participate in the design of PCDs, which could be locally made and used to store rabies vaccine for extended periods. At the end of the workshop five PCD designs were selected by the delegates for prototype development and testing. Following the workshop, local product designers, with the required skills to manufacture the PCDs, were recruited to build a prototype of each design.

### 2.2. Phase II: Field Testing

**Figure 1:**
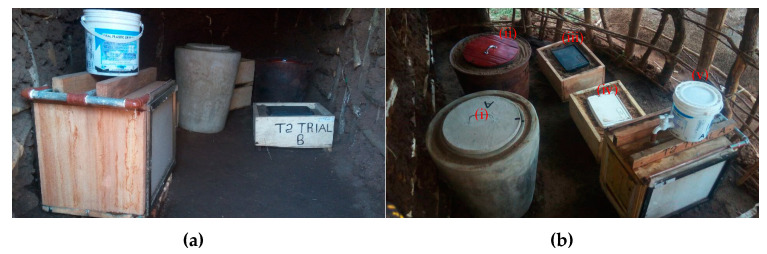
Passive cooling device (PCD) designs stored indoors (**a**) and outdoors (**b**). The different PCDs are labeled as follows: (i) Zeepot cementsand, (ii) Zeepot Clay, (iii) Coolbox sand, (iv) Coolbox sawdust and (v) Coolgardie.

Field testing of the five prototype PCDs, shown in Figure 1, was carried out in Bonchugu village in the Serengeti district. A locally typical house was selected as the venue for testing. The house was divided into two equal compartments, the first a closed area with walls that completely sealed the room (indoor), and the second a porous compartment with open walls (outdoor) as shown in Figure 1. An identical set of prototypes was placed in the indoor and outdoor areas. Each prototype was equipped with a digital temperature data logger (Sensormetrix, London, UK, www.sensormetrix.co.uk) configured to record the temperature inside the prototype at intervals of three hours over a two-week trial period. The data loggers were placed inside the inner units (cooling compartments) of the PCDs. In addition, the peak ambient temperature was recorded each day with another logger placed in the shade outside the trial house. The performance of each PCD was evaluated by comparing the temperature means and ranges of the prototypes. Similarly, the performance of each prototype in the indoor and outdoor areas was compared. Selection criteria for the next phase of the study were the ability of the prototype to maintain storage temperatures below 30 °C with minimal variation, the cost of PCD production, storage capacity, cooling media replenishment frequency and the prototype lifespan. The lifespan of each prototype was estimated by the developers judged from their experience of making similar products.

### 2.3. Phase III: Refinement

The two prototype designs that were selected during Phase II (field testing) were improved using feedback provided by the field-testing team and then subjected to a further two-month period of testing. Data analysis and performance appraisal of each prototype were undertaken according to the previously described criteria. At the end of Phase III, a single prototype was selected to proceed to Phase IV (final testing).

### 2.4. Phase IV: Final Testing

The performance of the selected prototype was evaluated over a 12-month period in a single location. The selected PCD was placed within a typical building with temperature loggers inside the PCD to record internal temperatures and on an internal building wall to record ambient temperatures at 3-h intervals. Further to the 12-month test, the performance of the selected prototype was also tested in three separate sites with different weather conditions for two months, during the warm season from 4 March to 3 May 2019. The selected sites represented a range of temperature environments. Sirari village in the Tarime district typically experiences relatively low temperatures with an average temperature during the final testing period of 21 °C; Mugumu town in the Serengeti district experiences more moderate temperatures (average temperature 24 °C) and Bokore village in the Bunda district experiences higher ambient temperatures (average temperature 26 °C) based on recent data from the Tanzania Meteorological Authority website (http://www.meteo.go.tz/). Descriptive statistics were used to analyze the logged temperature data. The null hypothesis that the average temperatures across the different prototypes were equal was tested using ANOVA. Time series and boxplots were used to examine temperature trends and variability. All data were analyzed using the R version 3.5.3 statistical computing language [17].

## 3. Results

### 3.1. Phase I: PCD Design and Development

The five novel PCD designs that were selected for prototype development during the workshop and the ranking they received are shown in Table 1.

**Figure 2:**
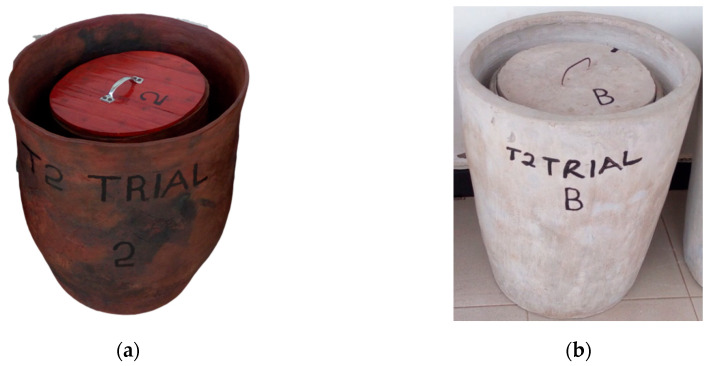
Second generation Zeepot (**a**) Clay and (**b**) Cementsand prototypes manufactured and tested during the refinement phase.

Pot-in-Pot systems: The system that delegates ranked the highest was a ‘pot-in-pot’ system (consisting of an inner pot nested within a larger outer pot) that exploited the evaporation of water as a cooling mechanism. The pot-in-pot system included a middle compartment filled with absorbent materials (multilayer) providing an effective barrier against heat exchange between the external surroundings and the cooling compartment. The design was named a ‘Zeepot’ after a traditional food storage pot commonly used in Africa to keep vegetables fresh. Two different Zeepot designs were suggested for development, (i) Zeepot Clay and (ii) Zeepot Cementsand. The two Zeepot prototypes were similar in design, however the Zeepot Clay was made from clay soil whereas the Zeepot Cementsand was made from cement and sand (Figure 2). Briefly, to construct the Zeepot Clay, the potter started by mining clay soil, which was then mixed with water to form clay. The potter punched the mass of clay to create a hole in the center, with the final shape formed by spreading apart the clay. The lid for the device was prepared using wooden materials. The pot was left for 2 days to dry in direct sunlight before being fired for 3–4 h. To manufacture the Zeepot Cementsand, cement and sand were mixed in the ratio 1:3, then water added to form mortar. A scoop was used to place the mortar outside of a conical shaped cylinder mold to create the shape and structure of the Zeepot Cementsand. Cylinder molds of different sizes were used for the inner and outer pots. The pots were filled with water and kept at room temperature for 3–4 days to complete the curing process.

Cool Box systems: Two cool box system prototypes (Coolbox-sand and Coolbox-sawdust) were selected for development. The cool box systems were constructed using ready-made cool boxes (Princeware) sold in local shops placed inside a rectangular wooden box. Sand or sawdust was added into the compartment between the constructed box and the cool box to form Coolbox-sand and Coolbox-sawdust, respectively (Figure 1).

Coolgardie system: This system was manufactured using a combination of the constructed wooden box lined with cloth. A five-liter plastic bottle was placed on top of the box with two drainage pipes connecting the bottle and the cloth (Figure 1). The Coolgardie system required replenishment of water every 3 h.

In general, it took approximately 4–7 days to complete the manufacture of each prototype. Costs of production included the labor charges and purchase of materials needed for the construction of each prototype.

### 3.2. Phase II: Field Testing and Selection

The temperatures recorded within the candidate prototypes during the field testing and selection phase are given in Table 2. The internal temperature range of all five prototypes that were stored indoors ranged from 15.5 to 21.6 °C, whilst the internal temperature range of the same prototypes stored in the outdoor area was wider, 15.1–24.3 °C. This suggests that storing the PCDs inside a building with solid walls was preferable. Across the five PCD prototypes, the Coolgardie PCD stored indoors had the lowest mean internal temperature (18.2 °C). However, because the Coolgardie required a continuous supply of water, its use was determined to be too costly and this design was not considered for the next phase. Excluding the Coolgardie, the design with the lowest mean internal temperature was the Zeepot Clay with 18.6 °C. Relative to the other prototypes investigated, the Zeepot Clay also had the narrowest temperature variation (standard deviation of 0.8 vs. 1.2 for the Coolgardie stored indoors). Although the Coolbox Sand had the next lowest internal temperature it was not selected for further development due to its low storage capacity. The Zeepot Cementsand had a mean temperature 0.7 °C higher than the Zeepot Clay, was constructed with hardy materials and was considered to have a longer lifespan than the Zeepot Clay. Due to this perceived robustness, the Zeepot Cementsand was also selected to proceed to Phase III (refinement) so that its performance could be further evaluated.

### 3.3. Phase III: Refinement

The Zeepot PCD design improvements from Phase II (field-testing) included increasing the volume from a storage capacity of 3.7 L (allowing storage of 40 vaccine vials) to 23.4 L (allowing storage of 550 vials), strengthening the external clay wall and redesigning the lid seals to fit more tightly (Figure 2).

**Figure 3:**
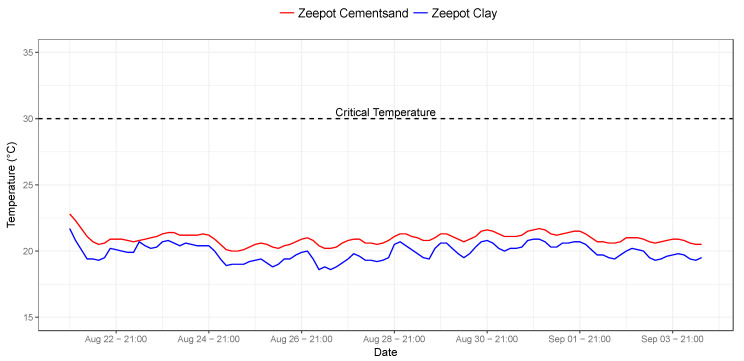
Time series showing the internal temperatures of the improved Zeepot Clay (blue line) vs. Zeepot Cementsand (red line) designs. The critical temperature above which the potency of thermotolerant rabies vaccines decrease is indicated by the dashed line.

Following these improvements, the second generation of Zeepot Clay and Zeepot Cementsand prototypes were manufactured and subjected to a two-month period of testing. The results from the testing are shown in Figure 3. The Zeepot Clay had a mean internal temperature of 19.9 °C (18.6–21.7 °C, SD = 0.6), whilst the Zeepot Cementsand had a mean internal temperature of 20.9 °C (20−22.8 °C, SD = 0.4). Both Zeepot prototypes maintained internal temperatures below 24 °C, however, because temperatures were consistently lower within the Zeepot Clay, this PCD prototype was selected to proceed to Phase IV (final testing).

### 3.4. Phase IV Final Testing

**Figure 4:**
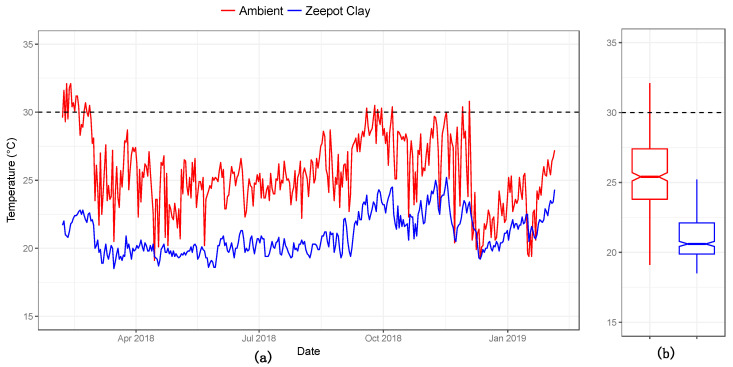
(**a**) Daily time series and (**b**) boxplot of ambient (red) and internal temperatures within the Zeepot Clay (blue) over the 12-month testing period in Bonchugu village, Serengeti district. The boxplot indicate median and range of temperatures whereas the dashed line shows maximum storage temperature above which the Nobivac^TM^ Canine Rabies vaccine loses potency.

The results from the 12-month trial conducted in Bonchugu village, Serengeti district are shown in Figure 4. The mean ambient temperature was 25.5 °C (19.1–32.1 °C, SD = 2.6), whilst the mean temperature within the Zeepot Clay was 21.0 °C (18.3–25.2 °C, SD = 1.5). Thus, despite ambient temperatures fluctuating by more than 10 °C during the year and maximum temperatures exceeding 32 °C, the temperature inside the Zeepot Clay remained relatively constant and well below the critical temperature, at which potency declines, of 30 °C.

**Figure 5:**
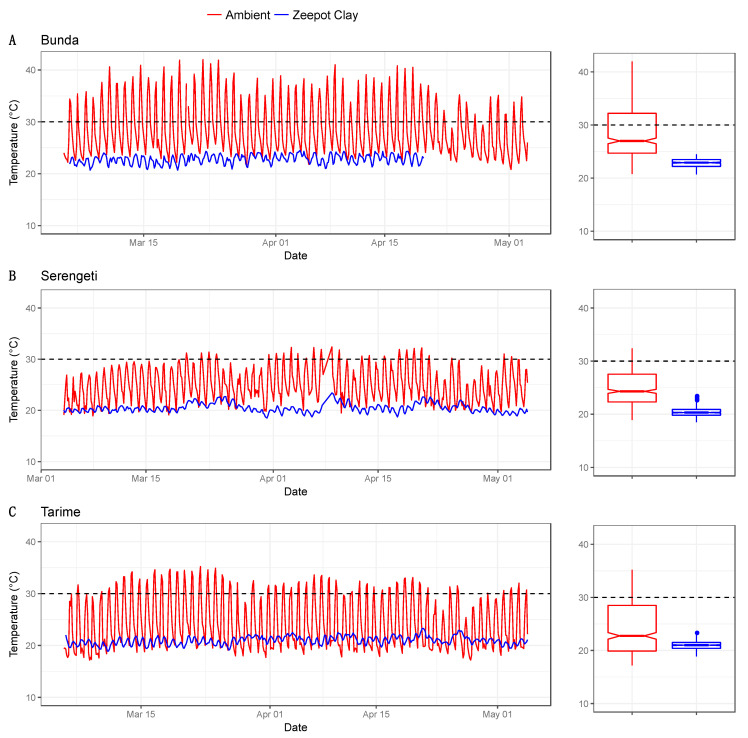
Ambient vs. internal storage temperatures within the Zeepot Clay over a 2-month period in three sites (**A**–**C**). Time series and boxplots indicate the median and range of temperatures in the three sites. The maximum storage temperature above which the Nobivac^TM^ Canine Rabies vaccine loses potency is indicated by the dashed line.

The internal temperatures of each Zeepot Clay and the ambient temperatures across the three locations over the two-month testing period are summarized in Table 3 and performances compared in Figure 5. The highest ambient temperature was recorded in Bunda (43.5 °C), whilst the lowest was recorded in Tarime 17.2 °C. At the time of the highest ambient temperature, the temperature inside the Zeepot Clay was 22.4 °C (Figure 5A).

The mean ambient temperature across the three sites was 26.0 °C, with a range of 17.2–43.5 °C, while the mean temperature inside the Zeepot Clay across the three sites was 21.4 °C, with a range of 20.4–22.8 °C. The logger used in the Bunda district stopped recording the temperature inside the Zeepot Clay just before the end of the trial period (Figure 5A). The slight increase in the internal temperature of the Zeepot Clay deployed in Serengeti (Figure 5B) between 7 and 10 April could be because the minimum ambient temperature was considerably higher during this period.

## 4. Discussion

A global target to eliminate human deaths from canine-mediated rabies has been set for 2030 [18]. For this goal to be reached, systems for the delivery of mass dog vaccination need to be operational in LMICs with endemic rabies [19]. To address this challenge, we embarked on a study to develop a practical and affordable PCD for storage of a thermotolerant rabies vaccine in areas with limited electricity. In total, five designs were investigated as potential prototypes for vaccine storage of which one, the Zeepot Clay, was selected as the best performing design. The internal temperature of the Zeepot Clay PCD remained below 26 °C over a 12-month testing period (maximum temperature of 25.2 °C), despite ambient temperatures exceeding 42 °C, with seasonal and geographical variation having minimal impact on performance. Temperature fluctuations can detrimentally affect vaccine potency [20], and so it was encouraging that the Zeepot Clay had the narrowest temperature variation of the prototypes. For example, when ambient temperature reached 43.5 °C, the Zeepot Clay internal temperature was just 22.4 °C (Figure 5A). We speculated that the low relative humidity in the Zeepot Clay accelerated evaporation thereby cooling the inner compartment of the device [21]. Our results indicate that the Zeepot Clay could be used for thermotolerant vaccine storage in places where refrigeration is not available. With respect to rabies specifically, we find that the locally produced Zeepot Clay is both affordable and effective and we suggest that it can support the scaling-up of mass dog rabies vaccination campaigns in areas with limited electricity across Tanzania and potentially elsewhere with similar environmental conditions where cold-chain storage of dog vaccines is challenging.

Commercial PCDs have been used in the public sector to store vaccines for tuberculosis, polio, influenza, whooping cough, tetanus, hepatitis B and diphtheria. For example, the Arkteks^®^ PCD was used to deliver vaccines during the 2014–2015 Ebola outbreak in West Africa and in Nepal to assist in the immunization campaign after the 2015 earthquake [22,23]. However, despite their utility, the price of commercially manufactured PCDs is often very high, ranging from US$700 to 3000 per unit [15]. Some designs require substantial maintenance, for example the Arkteks^®^ and the Nano-Q^®^ PCDs whilst able to keep temperatures inside the vaccine compartment at refrigeration temperature for many days, require recharging with ice. The Zeepot Clay is not designed to maintain refrigeration temperatures, but instead is capable of maintaining temperatures below levels at which thermotolerant vaccines such as the Nobivac^TM^ Canine Rabies vaccine begin to lose potency (30 °C). At US$11 per unit, the Zeepot Clay is considerably less expensive than commercial PCDs and is made of a material that is readily available in many areas across Sub-Saharan Africa and Asia (clay soil), making the Zeepot Clay feasible to manufacture locally at low cost.

Other factors for consideration in the design and use of PCDs are their maintenance demands. All commercially available PCDs require prior cooling through use of ice or placement inside refrigeration units, and as such none are able to keep vaccines cool for extended periods without power when ambient temperatures are high. For example, the Apex™ Vaccine Carrier Box AIVC 44, which costs US$9.16 and has a storage capacity of 1.67 L, uses ice packs to keep the vaccine compartment at refrigeration temperature for up to 35.7 h. The protocol for maintenance of the Zeepot Clay is inexpensive and simple, requiring only 1.5 L of water to be added as a coolant once every other day, making it straightforward to use with minimal training required. Indeed, in our study livestock field officers received 1-h on-site training and were considered proficient to take care of the device. These features suggest that the Zeepot Clay might be an affordable and logistically attractive option for storing thermotolerant rabies vaccines for extended periods in remote settings. 

The Zeepot Clay is suitable for storing vaccines in one place, whilst smaller passive containers, such as plastic cold boxes, are required to transport smaller batches of vaccine during mass dog vaccination campaigns from the storage location to the point of use. Cold water-packs or bottles of drinking water can be added to keep the temperature within the smaller containers relatively cool for the period required in the field. 

We did not measure the durability and lifespan of the PCD devices. Rather, the lifespan of each prototype was estimated by the product designers based on their knowledge and experience of how durable the prototype materials typically are in the local environment. Further study to investigate the durability of the Zeepot Clay would be useful.

PCDs have been used in public health programs that have protected millions of people against vaccine-preventable diseases [24]. Through its ability to store thermotolerant rabies vaccines for extended periods, we suggest that the Zeepot Clay PCD can enable penetration of mass dog vaccination campaigns to hard-to-reach communities in areas with limited cold chain, benefitting national governments planning to scale-up rabies elimination programs. If thermotolerance can be demonstrated in other canine rabies vaccines (as expected) or in vaccines used for other vaccine preventable diseases that affect communities living in areas with limited cold-chain, the utility of the Zeepot Clay or similar locally made PCDs could be even greater. We also anticipate that this tool could be used to store other thermotolerant vaccines, however more work is required to confirm this. Community engagement in the development process and the ability to produce this tool locally will likely increase acceptability and uptake.

## 5. Conclusions

Ongoing innovations in the development of PCDs have great potential to address the challenge of vaccine storage for last-mile immunization. Our PCD prototype, the Zeepot Clay, consistently maintained temperatures below 26 °C even when ambient temperatures exceeded 40 °C. This suggests that, under proper care and monitoring, the tool can be used to store rabies vaccine in areas without an electric grid for up to three months without compromising vaccine potency. Storing small batches of vaccines (300–500 vials) in PCDs such as the Zeepot Clay in a ‘decentralized’ manner across remote landscapes could potentially allow vaccination efforts to reach many more dogs than is currently achieved through traditional ‘centralized’ approaches. Given that sustaining high levels of vaccination coverage is a critical component of any successful vaccination campaign, this could be of great importance as we strive to eliminate dog-mediated human rabies globally by 2030.

## Figures and Tables

**Table 1:** PCD models developed by local designers and used in the first round of testing.

Design Name	Zeepot Clay	Zeepot Cementsand	Coolbox Sand	Coolbox Sawdust	Coolgardie
Shape	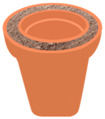	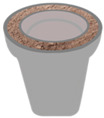	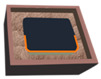	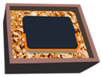	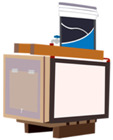
Materials	Clay	Cement, sand	Wood, coolbox (Princeware), 6ltrs	Wood, coolbox (Princeware), 6ltrs	Wood, fabric, plastic
Manufacturing time (Days)	7	7	4	4	5
Production Cost (US$)	11	30	17	17	26
Absorbent	Sand	Sand	Sand	Sawdust	Fabric
Cooling media	Water	Water	Water	Water	Water
Estimated lifespan (years)	7	>10	2	2	1
Ranking	1	4	2	3	5

**Table 2:** Summary of the temperatures recorded within each PCD prototype during the initial 2-week testing period.

Prototype	Location	Temperature Range in °C	Mean Temperature (SD)
Zeepot Clay	Indoor	16.6–20.5	18.6 (0.8)
	Outdoor	16.5–22.0	19.1 (1.1)
Zeepot Cementsand	Indoor	16.9–21.6	19.3 (0.9)
	Outdoor	16.4–22.0	19.6 (1.2)
Coolbox sand	Indoor	16.5–21.4	18.9 (1.0)
	Outdoor	16.4–24.3	20.1 (2.1)
Coolbox sawdust	Indoor	16.8–21.5	19.3 (1.0)
	Outdoor	16.5–23.4	20.0 (1.8)
Coolgardie	Indoor	15.5–20.7	18.2 (1.2)
	Outdoor	15.1–22.7	18.9 (1.9)

**Table 3:** The mean ambient temperature and internal temperature of the Zeepot Clay across three sites in the Mara region.

Site (Village, District)	Prototype	Temperature Range in °C	Mean Temperature (SD)
Bokore, Bunda	Zeepot Clay	20.7–24.5	22.8 (0.9)
	Ambient temperature	20.8–43.5	28.7 (5.0)
Sirari, Tarime	Zeepot Clay	18.9–23.3	21.0 (0.8)
	Ambient temperature	17.2–35.2	24.3 (4.9)
Mugumu, Serengeti	Zeepot Clay	18.5–23.4	20.4 (0.9)
	Ambient temperature	18.9–32.4	24.9 (3.3)
